# Endoscopic versus open carpal tunnel release: A short-term comparative study

**DOI:** 10.4103/0019-5413.30527

**Published:** 2007

**Authors:** R Malhotra, E Krishna Kiran, Aman Dua, S G Mallinath, S Bhan

**Affiliations:** Department of Orthopedics, All India Institute of Medical Sciences, New Delhi, India

**Keywords:** Carpal tunnel syndrome, endoscopic carpal tunnel release

## Abstract

**Objective:**

To compare the results of endoscopic carpal tunnel release (CTR) with open CTR in patients with idiopathic Carpal tunnel syndrome (CTS).

**Materials and Methods:**

Seventy-one patients with CTS were enrolled in a prospective randomized study from May 2003 to December 2005. All patients had clinical signs or symptoms and electro-diagnostic findings consistent with carpal tunnel syndrome and had not responded to nonoperative management. Sixty-one cases were available for follow-up. Endoscopic CTR was performed in 30 CTS patients and open CTR was performed in 31 wrists (30 patients). Various parameters were evaluated, including each patient's symptom amelioration, complications, operation time, time needed to resume normal lifestyle and the frequency of revision surgery. All the patients were followed up for six months.

**Results:**

During the initial months after surgery, the patients treated with the endoscopic method were better symptomatically and functionally. Local wound problems in terms of scarring or scar tenderness were significantly more pronounced in patients undergoing open CTR compared to patients undergoing endoscopic CTR. Average delay to return to normal activity was appreciably less in group undergoing endoscopic CTR. No significant difference was observed between the endoscopic CTR group and open CTR group in regard to symptom amelioration, electromyographic testing and complications at the end of six months.

**Conclusion:**

Short-term results were better with the endoscopic method as there was no scar tenderness. Results at six months were comparable in both groups.

The carpal tunnel syndrome (CTS) is a common pathology, recognized since one and a half century. Carpal tunnel syndrome (CTS) is caused by compression of the median nerve at the wrist resulting in hand numbness, loss of dexterity, muscle wasting and decreased functional ability at work. Open Carpal tunnel release (CTR) has been considered the operative procedure of choice for decompression of the median nerve at the wrist in patients who have idiopathic CTS.^1^–^3^ Recently, there has been a trend to treat CTS by the endoscopic release of the transverse carpal ligament.^4^,^5^ Endoscopic carpal tunnel release (ECTR) is claimed to be associated with minimal pain and scarring due to small incision, a shortened recovery period and a high level of patient satisfaction.^6^ Current literature suggests that the long-term results of endoscopic CTR are the same as those of open CTR.^7^ However, there are some reports that doubt the claims that the endoscopic carpal tunnel release is associated with quicker functional recovery and less postoperative pain.^8^ Concerns persist with the possibility of endoscopic release resulting in incomplete release, higher rate of recurrence along with questionable safety of endoscopic techniques, cost of endoscopic equipment and training and difficulty of the surgery.^1^,^4^

We conducted a randomized, prospective study to investigate whether early and late recovery after open CTR is comparable with endoscopic carpal tunnel release. The clinical as well as electrophysiological assessment of the early as well as late recovery was made following both the surgical procedures and the results were compared. We also compared the rate of recurrence, the need for revision surgery and the incidence of complications following the two procedures.

## MATERIALS AND METHODS

Seventy patients (71 wrists) with CTS were randomly chosen for a prospective study from May 2003 to December 2005. *Randomization was done using ‘Sealed envelope’ technique.* Patients with CTS who failed with conservative treatment with splinting and nonsteroidal anti-inflammatory medications for a period of three months were included in the study. The diagnosis of CTS was based on at least two notable findings by history and examination (such as night pain, median nerve sensory disturbances, Phalen's test or Tinel's sign at the wrist). Due to low sensitivity and specificity associated with the clinical tests the diagnosis was confirmed by electrophysiological studies. Patients with inflammatory conditions, concomitant pregnancy, patients on anticoagulants or with bleeding or coagulation disorders, patients on hemodialysis and patients with previous hand trauma were excluded. Preoperatively grip strength and pinch strength were recorded. Electrophysiological study was performed preoperatively to confirm the diagnosis, postoperatively at the end of one month to compare the early recovery and was repeated at the end of six months for long-term benefit. Electrophysiological confirmation was established with use of the combined sensory index, which is the sum of three latency differences: median-ulnar across the palm (palmdiff), median-ulnar to the ring finger (ringdiff) and median-radial to the thumb (thumbdiff).^9^ All patients included in the study met the American Association of Electrodiagnostic Medicine diagnostic criteria for CRS.^9^,^10^

### Endoscopic group

There were 36 patients in this group. Out of these 30 patients were available for follow-up. The mean age was 44.6 years and the dominant hand was involved in 23 patients. Twelve of the patients were female. The mean duration of symptoms was 5.1 months (range, 4-10 months). Twelve patients had been treated with a splint for six weeks prior to the surgery. Three patients had been treated with a steroid injection.

### Open-release group

There were 34 patients (35 wrists) in this group. Out of these, 30 patients (31 wrists) were available for follow-up. One patient had a bilateral wrist involvement. The mean age was 45.3 years and the dominant hand was involved in 22 patients. Twenty-three of the patients were female. The mean duration of symptoms was 6.5 months (range, 3-12 months). Ten patients had been treated with a splint for six weeks prior to the surgery. Seven patients had been treated with a steroid injection prior to the surgery.

### Surgical technique

All surgical procedures were performed under the tourniquet control. Patient preference for general anesthesia or regional anesthesia was accommodated. A single portal endoscopic carpal tunnel release was performed in the first group (30 cases) and a short incision open carpal tunnel release was done in the second group (31 cases).

### Endoscopic CTR

A 1.0 cm transverse incision is made at the level of the distal wrist crease in the center of the volar aspect of the wrist. The incision is centered over the palmaris longus if it is present. The palmaris longus is retracted radially to protect the palmar cutaneous branch of the median nerve. Scissors are used to make a distally based flap in the flexor retinaculum. The median nerve is identified deep to the retinaculum and a synovial elevator is used to reflect the synovial tissue from the undersurface of the transverse carpal ligament. Dilators are used to provide a space for the device. The device is inserted to a depth of <3.0 cm to avoid injury to the superficial palmar arch or the common digital nerve to the fourth web space. Once the device is in place, its trigger is depressed to elevate the blade and then the device is withdrawn to release the transverse carpal ligament. Several passes may be required when the transverse carpal ligament is very thick. The incision is closed with monofilament sutures.

### Open CTR

The incision is made 2 mm ulnar to the thenar crease, just distal to the Kaplan oblique line and extended 3.0 to 4.0 cm proximally toward the distal wrist crease. The superficial palmar fascia, transverse carpal ligament and antebrachial fascia are divided. The tourniquet is deflated after the wound is closed with monofilament sutures. Neither tenosynovectomy nor neurolysis was performed in this group.

Postoperatively, we used a bulky soft dressing covering the wrist and hand. This dressing worked as good as a splint. This dressing was kept in place till suture removal at the end of two weeks. After this patients were started on active assisted exercises for a period of two to four weeks followed by passive exercises and normal activity.

### Postoperative evaluation

Postoperative evaluation was done at one month and six months after the surgery. Early recovery and completeness of recovery were assessed. Recording was made of the improvement in symptoms, function and electrophysiological studies and the complications of the surgical procedure. Symptoms were evaluated by eliciting the severity of incisional pain, changes in severity of pain, tingling sensations, severity of nighttime numbness and hand weakness. Function was evaluated by grip strength and pinch strength and compared with preoperative values. The time taken to resume the daily activities was recorded. Any residual pain or scar tenderness was elicited.

## RESULTS

### Preoperative parameters [Table 1]

**Table 1:** Preoperative parameters of the endoscopic release group (1) and the limited open release group (2)

Parameter (preoperative)		Group 1 n=30	Group 2 n=31
Hand involvement	R	21	17
	L	9	12
	B/L	-	1
Duration of symptoms	3-6 m	18	15
	6-9 m	12	14
	> 9 m		2
Pain severity (1-10)	0-3	-	-
	4-6	20	18
	7-10	10	13
Numbness / paresthesia	Mild	-	-
	Moderate	24	23
	Severe	6	8
Sensory loss		24	27
Wasting of APB		3	7
Previous treatment	Analgesics	30	31
	Local steroids	3	7
	Splints	12	10
	Open surgery	-	-
	Arthroscopic surgery	-	1
Initial response to previous treatment	Excellent	5	4
	Good	17	12
	No response	8	15
	Worsening	-	-
Overall response to pervious treatment	Excellent	-	-
	Good	-	-
	No response	30	29
	Worsening	-	2
ADL affected due to CTS	Mild	7	3
	Moderate	19	10
	Severe	4	18

APB - Abductor pollicis brevis, CTS - Carpal tunnel syndrome

During three years of the study 70 patients (71 wrists) with carpal tunnel syndrome were operated by either endoscopic or open technique and 60 patients (61 wrists - 30 operated by endoscopic release and 31 by open release) were followed at one month and six months. The preoperative parameters [Table 1] were comparable in both the groups. The average duration of symptoms prior to surgery was 5.1 months in Group 1 and 6.5 months in Group 2. Nearly all patients had moderate degree of paresthesia and sensory loss. Wasting of abductor pollicis brevis was present in three patients in Group 1 and seven patients in Group 2.

### Postoperative parameters: 1 month

Onset of relief of symptoms was within three days in 17 out of 30 patients who underwent endoscopic carpal tunnel release whereas 14 wrists out of 31 undergoing open carpal tunnel release reported early relief. Four patients in Group 2 reported onset of pain relief three weeks following surgery. Nineteen out of 30 patients in Group 1 reported nearly complete remission of symptoms while 17 patients out of 31 showed the same in Group 2. The incidence of local pain and scar tenderness was significantly higher in Group 2, where 20 wrists out of 31 reported mild local pain and 19 patients reported scar tenderness as compared to only three patients who reported local pain in Group 1. Local wound hematoma was noticed in one patient in Group 2. Hematoma resolved within two weeks with compressive dressings. No complications in the form of neurovascular injury or tendon injury were observed. All patients had complete alleviation of night symptoms and no patient reported worsening of symptoms or new development of sensory loss.

### Postoperative parameters: 6 months [Table 2]

**Table 2:** Postoperative parameters of the endoscopic release group (1) and the limited open release group (2) at 6 months

Parameter		Group 1 n=30	Group 2 n=31
Relief in pain (pain scale-0-10)	0-3	28	29
	4-6	2	2
	7-10	-	-
Recurrence of symptoms		-	-
Local scarring		-	2
Keloid formation		-	1
Remission of symptoms	100	23	20
	> 75% improvement	6	8
	50-75% improvement	1	2
	< 50% improvement	-	-
Residual numbness		2	4
Residual motor weakness		2	5
Subjective improvement	Excellent	25	21
	Good	5	9
	No improvement	-	-
	Worsening	-	-
Scar tenderness (+)		-	9
Time taken to return to daily activities	Average	16 days	20 days

Sixty-one cases (30 in the endoscopic release group and 31 in the open release group) were available for follow-up at six months postsurgery. Twenty-three patients out of 30 in Group 1 showed near complete relief of symptoms, while 20 wrists out of 31 showed the same in Group 2. Local scarring and Keloid formation was observed in four and one patients respectively in Group 2. Residual numbness was observed in two patients in Group 1 and 4 four patients in Group 2. Residual motor weakness was observed in two patients in Group 1 and five patients in Group 2. The higher incidence of residual motor weakness or numbness in Group 2 could be due to preexisting neurological deficit in these patients. At six months scar tenderness was observed in nine patients of Group 2. No incision-site-related complication was observed in endoscopic CTR group. Average duration to return to daily activities was 16 days for Group 1 patients whereas the delay in return to normal activities was longer in Group 2 patients (average 20 days).

At six months follow-up grip strength improvement was observed in both the groups. In the endoscopic release group the grip strength improved from a preoperative mean of 19.9 kg to a postoperative mean of 22.8 kg. Comparable results were observed in the open release group where grip strength improved from a preoperative value of 19.2 kg to a postoperative value of 22.2 kg.

### Electrophysiological results [Tables 3 and 4]

**Table 3:** Table 3: Electrophysiological Data: Endoscopic Carpal tunnel release group

Parameter	Baseline (mean)	1^st^ Visit (mean)	2^nd^ Visit (mean)
Motor nerve- Affected median			
DL (ms)	4.7	3.9	3.7
CMAP Amp (mV)	4.7	5.3	5.4
NCV (m/s)	40	50	50
Motor nerve- Contralateral median			
DL	3.3	3.2	3.2
CMAP amp	5.5	5.6	5.5
NCV	50	50	50
Sensory- Affected median			
DL	4.1	3.2	3.2
SNAP amp (mV)	9	12	13
NCV	42	48	48
Sensory- Contralateral median			
DL	2.1	2.2	2.2
SNAP amp	15	15	15
NCV	50	50	50
Sensory- Right ulnar			
DL	1.7	1.7	1.7
SNAP amp (mV)	25	25	25
NCV	49	49	50
MP latency	2.5	2.5	2.5
MP amplitude	6.5	6.5	6.5
Sensory- Left ulnar			
DL	1.9	1.9	1.9
SNAP amp	27	27	27
NCV	51	51	51
MP latency	2.4	2.4	2.4
MP amplitude	6.3	6.3	6.3

DL: Distal latency, CMAP: Compound muscle action potential, NCV: Nerve conduction velocity, SNAP: Sensory nerve action potential, MP

**Table 4:** Electrophysiological data: Open Carpal tunnel release group

Parameter	Baseline (mean)	1^st^ Visit (mean)	2^nd^ Visit (mean)
Motor nerve- Affected median			
DL (ms)	4.8	4.0	4.0
CMAP amp (mV)	4.6	5	5.1
NCV (m/s)	40	47	48
Motor nerve- Contralateral median			
DL	3.1	3.1	3.1
CMAP amp	5.5	5.6	5.5
NCV	50	50	51
Sensory- Affected median			
DL	4.0	3.3	3.1
SNAP amp (mV)	10	13	15
NCV	39	49	50
Sensory- Contralateral Median			
DL	2.2	2.2	2.1
SNAP amp	16	16	16
NCV	51	51	52
Sensory- Right ulnar			
DL	1.7	1.8	1.8
SNAP amp (mV)	25	25	25
NCV	50	49	50
MP latency	2.3	2.3	2.2
MP amplitude	6.6	6.6	6.6
Sensory- Left ulnar			
DL	1.8	1.8	1.8
SNAP amp	26	25	25
NCV	50	50	50
MP latency	2.3	2.3	2.5
MP amplitude	6.7	6.7	6.7

DL: Distal latency, CMAP: Compound muscle action potential, NCV: Nerve conduction velocity, SNAP: Sensory nerve action potential

At one month after the surgery, the distal latency (both motor and sensory) of the median nerve across the wrist was reduced and the nerve conduction velocity (both motor and sensory) was increased in all the patients in both the groups.

In the endoscopic CTR group the average distal latency and conduction velocity recorded in the preoperative period was 4.7 ms and 40 m/s respectively. Six months postsurgery both the parameters improved to an average of 3.7 ms and 50 m/s respectively.

In the open carpal tunnel release group the average distal latency and conduction velocity recorded in preoperative period was 4.8 ms and 40 m/s respectively. Six months postsurgery both the parameters improved to an average of 4.0 ms and 48 m/s respectively.

However, no appreciable difference could be noted in the pattern of recovery between the two groups.

### Complications

Symptoms consistent with reflex sympathetic dystrophy, with swelling, redness and increased sweating, developed in two patients in the open release group. In one, the symptoms were mild and resolved after a brief course of physical therapy. In the other patient, the symptoms were more protracted and a regular therapy program as well as the use of nortriptyline was required.

Postoperative parameters of the two groups are presented in Table 2.

## DISCUSSION

Open CTR has been considered the operative procedure of choice for decompression of the median nerve at the wrist in patients who have idiopathic CTS.^2^,^3^ However, some authors have suggested that persistent weakness, tenderness of the scar and pain in the thenar or hypothenar area (pillar pain) occur frequently after open procedures.^11^–^15^ Endoscopic release of the carpal tunnel was introduced as an alternative method in the hope of decreasing the rate of these complications.^4^ Endoscopic CTR is claimed to be associated with minimal pain and scarring due to minimal incision, a shortened recovery period and a high level of patient satisfaction.^6^ Analysis of the outcomes of our study demonstrates that the patients who had undergone endoscopic release had greater relief of symptoms, improvement in function and satisfaction for the first three months following the surgery. Furthermore, they had faster recovery of both grip and pinch strength, findings that agree with those in the nonrandomized study performed by Palmer *et al*.^8^ We presume that a major factor in this regard is the fact that the palmaris brevis muscle and the palmar fascia are not divided with the endoscopic technique. The safety of the endoscopic technique has been a major concern. Although one isolated report focused on the risks involved in endoscopic surgery,^16^ these findings were not borne out in larger, prospective, multicenter trials.^4^,^8^,^17^ A success rate of 93.3% has been reported in 116 wrists of 84 patients followed for five years after endoscopic release surgery and the recurrence rate was only 0.96%.^17^

Reported complications after endoscopic carpal tunnel release include median nerve laceration, ulnar nerve laceration, vessel lacerations and tendon lacerations, ulnar neurapraxia and intense pain in the middle and ring fingers.^18^,^19^ Apart from the structure injury (nerve, vessel, tendon), recurrent hematoma and infection are the other complications reported after endoscopic carpal tunnel release. No complications occurred with the endoscopic technique in our study. The factor that we think reduced the rate of complications was a team that was familiar with fiberoptic-assisted surgery.

## CONCLUSIONS

Short-term results were better with the endoscopic method as there was no scar tenderness and results at six months were comparable in both groups. There were no significant complications associated with any of the two methods of carpal tunnel release.

## References

[1] Brown RA, Gelberman RH, Seiler JG, Abrahamsson SO, Weiland AJ, Urbaniak JR (1993). Carpal tunnel release. A prospective, randomized assessment of open and endoscopic methods. J Bone Joint Surg.

[2] Gelberman RH, Gelberman RH (1991). Carpal tunnel release. Open release of the transverse carpal ligament. Operative nerve repair and reconstruction.

[3] Pfeffer GB, Gelberman RH, Boyes JH, Rydevik B (1988). The history of carpal tunnel syndrome. J Hand Surg (Br).

[4] Agee JM, McCarroll HR, Tortosa RD, Berry DA, Szabo RM, Peimer CA (1992). Endoscopic release of the carpal tunnel: A randomized prospective multicenter study. J Hand Surg (Am).

[5] Chow JC (1990). Endoscopic release of the carpal ligament for carpal tunnel syndrome: 22-month clinical result. Arthroscopy.

[6] Trumble TE, Diao E, Abrams RA, Gilbert-Anderson MM (2002). Single-portal endoscopic carpal tunnel release compared with open release: A prospective, randomized trial. J Bone Joint Surg.

[7] Brief R, Brief LP (2000). Endoscopic carpal tunnel release: Report of 146 cases. Mount Sinai J Med.

[8] Palmer DH, Paulson JC, Lane-Larsen CL, Peulen VK, Olson JD (1993). Endoscopic carpal tunnel release: A comparison of two techniques with open release. Arthroscopy.

[9] Lew HL, Wang L, Robinson LR (2000). Test-retest reliability of combined sensory index: Implications for diagnosing carpal tunnel syndrome. Muscle Nerve.

[10] Practice parameter for electrodiagnostic studies in carpal tunnel syndrome: Summary statement (1993). American Association of Electrodiagnostic Medicine, American Academy of Neurology, American Academy of Physical Medicine and Rehabilitation. Muscle Nerve.

[11] Cseuz KA, Thomas JE, Lambert EH, Love JG, Lipscomb PR (1966). Long-term results of operation for Carpal tunnel syndrome. Mayo Clin Proc.

[12] Kulick ML, Gordillo G, Javidi T, Kilgore ES, Newmayer WL (1986). Long-term analysis of patients having surgical treatment for Carpal tunnel syndrome. J Hand Surg (Am).

[13] Kuschner SH, Brien WW, Johnson D, Gellman H (1991). Complications associated with Carpal tunnel release. Orthop Rev.

[14] MacDonald RI, Lichtman DM, Hanlon JJ, Wilson JN (1978). Complications of surgical release for Carpal tunnel syndrome. J Hand Surg.

[15] Seradge H, Seradge E (1989). Piso-triquetral pain syndrome after Carpal tunnel release. J Hand Surg (Am).

[16] Feinstein PA (1993). Endoscopic carpal tunnel release in a community-based series. J Hand Surg (Am).

[17] Agee JM, Peimer CA, Pyrek JD, Walsh WE (1995). Endoscopic Carpal tunnel release: A prospective study of complications and surgical experience. J Hand Surg (Am).

[18] Chung KC, Walters MR, Green field ML, Chernew ME (1998). Endoscopic versus open Carpal tunnel release: A cost-effectiveness analysis. Plast Reco nstr Surg.

[19] Muller LP, Rudig L, Degreif J, Rommens PM (2000). Endoscopic Carpal tunnel release: Results with special consideration to possible complications. Knee surgery.

